# The Use of Ketogenic Diet in Pediatric Patients with Epilepsy

**DOI:** 10.5402/2012/263139

**Published:** 2012-08-28

**Authors:** Amanda Misiewicz Runyon, Tsz-Yin So

**Affiliations:** Department of Pharmacy, Moses H. Cone Hospital, Greensboro, NC 27401-1020, USA

## Abstract

A ketogenic diet is a nonpharmacologic treatment strategy to control refractory epilepsy in children. Although this diet has been used successfully to reduce seizures since the 1920s, the anticonvulsant mechanism of ketosis remains unknown. The initiation of the diet requires an average four-day hospitalization to achieve ketosis in the patient as well as to provide thorough education on diet maintenance for both the patient and the caregivers. A ketogenic diet, consisting of low carbohydrate and high fat intake, leaves little room for additional carbohydrates supplied by medications. Patients on ketogenic diets who exceed their daily carbohydrate limit have the risk of seizure relapse, necessitating hospital readmission to repeat the diet initiation process. These patients are at a high risk for diversion from the diet. Patients admitted to the hospital setting are often initiated on multiple medications, and many hospital systems are not equipped with appropriate monitoring systems to prevent clinicians from introducing medications with high carbohydrate contents. Pharmacists have the resources and the expertise to help identify and prevent the initiation of medications with high carbohydrate content in patients on ketogenic diets.

## 1. Effect of Diet on Epilepsy

A ketogenic diet is a strict diet consisting of minimal carbohydrate and protein intake and increased fat intake. It is used as a nonpharmacologic mechanism to control intractable childhood epilepsy [1]. Ketogenic diets mimic the body's response to starvation by using fat as the primary energy source in the absence of an adequate dietary carbohydrate source. Under normal metabolism, the body metabolizes carbohydrates into glucose, the fastest source of energy for the body and typically the sole energy source for the brain. In a fasting state, amino acids cannot provide an adequate energy source for the brain and fatty acids cannot cross the blood brain barrier. The liver uses the fatty acids to make ketone bodies, which can cross the blood brain barrier and substitute for glucose as an energy source. The mechanism of how ketosis controls seizures is unknown; however, one theory is that ketones have an anticonvulsant effect when crossing the blood brain barrier. Regardless of the mechanism, the effects of ketosis on seizure control have been observed since this diet was introduced in the 1920s [1].

## 2. The Use and Effectiveness of Ketogenic Diets

The primary indication for a ketogenic diet is intractable childhood epilepsy. The treatment is typically recommended when traditional antiepileptic drugs (AEDs) have failed or AED therapy causes unacceptable side effects. Approximately 30% of children who develop epilepsy will develop refractory seizures unresponsive to pharmacologic treatment or experience intolerable side effects from antiseizure medications [2]. The International Ketogenic Diet Study Group, a panel of 26 pediatric epilepsy specialists and dieticians, published a consensus report agreeing that ketogenic diets should be strongly considered in a child who failed two to three anticonvulsant therapies, particularly in those patients with symptomatic generalized epilepsies [3].

### 2.1. Efficacy and Initiation

A 2006 meta-analysis [6] of 19 observational studies (1084 patients) found that after six months of initiating a ketogenic diet, approximately 60 percent of children had a greater than 50 percent seizure reduction and 30 percent had greater than 90 percent seizure reduction [6]. The results of the meta-analysis also suggest that children maintained on a ketogenic diet may also be able to reduce their AED with better seizure control. Children that benefited the most from the diet were those with generalized seizures and those between 1 and 10 years of age [6].

Studies investigating the effectiveness of ketogenic diets are all observational based and focus on the patients that were compliant with the diet; however, most of these studies have large dropout rates. In the above meta-analysis, about half of the patients dropped out. Families primarily discontinued the diet due to the lack of improvement in seizure control [6].

More recently, a randomized controlled trial was performed to test the efficacy of a ketogenic diet on drug-resistant childhood epilepsy [7]. The study included 145 children between 2 and 16 years of age who had at least daily seizures and had failed to respond to at least two antiepileptic drugs. Children were randomly assigned to receive a ketogenic diet immediately or to a control group, which initiated the diet 3 months after randomization. During the 3 months prior to the initiation of a ketogenic diet, the control group continued their normal diet without any dietary restrictions. The primary endpoint was a reduction in seizures at 3 months, and intention-to-treat analysis was used. At 3 months, the mean percentage of baseline seizures was significantly lower in the diet group than in the control group who had experienced an increase in seizures from baseline (62% versus 137%; *P* < 0.0001) [7]. In addition, 28 children in the diet group versus 4 children in the control group experienced a greater than 50% seizure reduction (*P* < 0.0001), and five children in the diet group had greater than 90% seizure reduction compared to zero children in the control group (*P* = 0.0582). Of the patients that dropped out from the study, only six patients were reported as being intolerant to the diet due to increased seizure frequency, extreme drowsiness, constipation, vomiting, or diarrhea [7]. One of the six patients who withdrew from the study developed hematuria secondary to renal debris, indicative of the risk of kidney stone formation while on a ketogenic diet [7].

Initiation of a ketogenic diet most often occurs in an inpatient setting at an epilepsy center in order to safely monitor glucose levels and urine ketone levels. Traditionally, the diet is initiated after a 24–48-hour fasting period, and it is slowly introduced until the patient successfully achieves the full ketogenic diet to be discharged home with [3]. The average hospital stay is four days, during which the family and the patient are educated on the diet. If ketosis is not maintained, the patient must return to the hospital to restart the entire diet initiation process; therefore, compliance with the diet is essential.

The compliance of the patients with the diet mainly depends on the types of diet and the patient population [3]. Children who are fed enterally usually demonstrate very high compliance rates, whereas a diet having a fat : nonfat ratio of more than 4.5 : 1 usually leads to poor compliance [3]. Older children and adolescent usually have difficulty adhering to strict diet ratio. Thus, a lower fat : nonfat ratio is often used in this population [3].

### 2.2. Types of Ketogenic Diets

Multiple variations of ketogenic diets exist, but the most commonly prescribed are the classic ketogenic diet, the modified Atkins diet, the low-glycemic index treatment diet, the medium-chain triglyceride (MCT) diet, and the modified MCT diet (Table 1) [4]. The classic ketogenic diet is the oldest of the diets and is one of the strictest of the diets. A gram scale is required to weigh food portions because no estimations are permitted. The diet restricts daily calories calculated by the patient's dietitian with a distribution of 85–90% long-chain fatty acid, 6–8% protein, and 2–4% carbohydrates [4].  Table 2 illustrates a sample calculation of daily energy requirements for this diet.

Unlike the standard Atkins diet, the modified Atkins diet does not restrict calories, allowing unlimited protein and fat intake, and is more lenient with the use of estimations of portion sizes. The modified Atkins dietary requirements are comprised of 60–70% long-chain fatty acid, 25–30% protein, and 5% carbohydrate [4]. The low-glycemic index (low-GI) treatment diet restricts the patient's carbohydrate intake to low-GI carbohydrates, allowing for a larger daily allowance of carbohydrates. The glycemic index scores individual carbohydrates based on each food item's effect on raising blood glucose within two hours of consumption. The diet's dietary distribution is 60–70% long-chain fatty acid, 20–30% protein, and 10% carbohydrate [4].

Normal dietary fat contains mostly long-chain triglycerides. Medium-chain triglycerides (MCTs), such as decanoic acid and octanoic acid, are absorbed more effectively and are more ketogenic than LCTs because they generate more ketones per unit of energy when metabolized. Patients on the MCT diet are able to introduce more carbohydrates and proteins in their diet compared to the classic ketogenic diet [4]. The MCT diet is comprised of 71% medium-chain fatty acid, 10% protein, and 19% carbohydrate. Alternatively, the modified MCT diet combines the use of both long-chain and medium-chain fatty acids. The modified MCT diet distributes the calories as 30% MCT oil, 40–50% conventional or long-chain fatty acids, 10–20% protein, and 5–10% carbohydrates [4]. The classical and modified MCT ketogenic diets are equally effective, and differences in tolerability are not statistically significant. Despite its flexibility, the MCT diet is disfavored since MCT oil is more expensive than other fats and is not covered by insurance companies [8].

### 2.3. Monitoring and Tolerability

The duration of the ketogenic diet varies among patients. The expected length of therapy should be discussed with the patient and/or the family prior to starting the diet, but most patients should expect a minimum of a 3-month trial period [3]. In regards to monitoring the effects of the diet, the anticonvulsant activity gradually increases over time but usually requires several days to weeks to see a noticeable effect. A six week treatment period is usually sufficient to determine success or failure. If seizure control is optimized after a few months, AED therapy may be tapered or discontinued. Monitoring urine ketones is necessary to ensure that the diet is being managed correctly, although the amount of urine ketones does not necessarily correlate directly with seizure control [1].

Ketogenic diets, like any other treatment, are not without risk and require monitoring of complications. Short-term adverse effects include dehydration, mild metabolic acidosis, and hypoglycemia during fasting [4]. Long-term adverse effects include nephrolithiasis, constipation, vitamin and mineral deficiencies, increased cholesterol, retarded growth in young children, and decreased bone mineral density [4]. Various laboratory values should be monitored initially and routinely (usually every 3 months for the 1st year) when patients are started on a ketogenic diet. Such laboratories include, but are not limited to, serum glucose, albumin, total protein, fasting cholesterol and triglycerides, and serum creatinine [3].

The addition of a ketogenic diet to a patient's current antiepileptic drug regimen is generally well tolerated and safe. There is some evidence demonstrating that the combination of a ketogenic diet with zonisamide is beneficial in reducing seizures [9]. Alternatively, children on phenobarbital have less success in managing seizures when a ketogenic diet was added [9]. There are a few drug interactions with ketogenic diets that prompt careful monitoring if the interaction cannot be avoided. In particular, monitor bicarbonate levels in patients on concomitant AED therapy consisting of a carbonic anhydrase inhibitor, such as acetazolamide or methazolamide. The reduction in bicarbonate levels in addition to the increased acid caused by ketones may cause metabolic acidosis [10].

## 3. Pharmacists' Involvement to Improve Outcomes in Patients on Ketogenic Diet

Pharmacists can play an important role in restricting the use of medications with high carbohydrate content. Ultimately, systems should be put in place to protect the patient from medications with high carbohydrate content in order to maintain ketosis and, thus, seizure control.

Table 3 contains a list of pediatric antiepileptic drugs and their respective carbohydrate contents [5]. Carbamazpine suspension, ethosuximide syrup, phenobarbital elixir, and valproic acid syrup contain the highest amounts of carbohydrate and should be avoided in ketogenic diet patients. Choosing the tablet or capsule formulation for each of these medications reduces the daily carbohydrate intake while still providing the same dose to the patient. A general rule of thumb is that carbohydrate content is the highest in suspensions and solutions, lower in chewable and disintegrating tablets, and lowest in tablets and capsules that are meant to be swallowed whole. Also, labels reading “sugar-free” can be misleading and often contain carbohydrates, such as sorbitol. The “sugar free” label is used primarily for diabetics and may contain carbohydrate-containing excipients which will not affect glycemia but might affect ketosis in the diet [11].

Tables 4 and 5 also contain a list of common pediatric antibiotics and antipyretics with their respective carbohydrate contents since these medications are frequently encountered medications in the pediatric population [5]. In order to illustrate how easily medications can alter a ketogenic diet, a patient case has been provided (see Box 1).

This patient example demonstrates the importance of monitoring the carbohydrate content in medications. Pharmacists have the resources and the expertise to help identify and prevent the initiation of medications with high carbohydrate content in patients on ketogenic diets. In the inpatient setting, one solution to help avoid the prescribing of high carbohydrate-content medications is to add “sugar” as an allergy to the patient's profile or medical record. For institutions utilizing electronic medical records, a pop-up screen alerting the clinician that the patient is “on a ketogenic diet” is another practical option. At our institution, pharmacists use VigiLanz Dynamic Monitoring System software (Minneapolis, MN) to help identify and prevent adverse drug events. Pharmacists at Cone Health can proactively identify patients on ketogenic diets by adding a new rule to the VigiLanz software, alerting pharmacists when pediatric patients have been admitted with a history of seizure plus an allergy to dextrose. Once the pharmacists have been alerted, they can follow the patient closely to recommend low carbohydrate-content medication alternatives to the treating physician team if the patients are on a ketogenic diet. Since patients on ketogenic diets may be admitted for nonseizure related issues, the diet may be overlooked during the treatment of the primary condition. Providing pharmacists with a system to catch high carbohydrate-content medications during order entry or via a software alert system can protect the patient from seizure recurrence and the reinitiation of a ketogenic diet.

## 4. Conclusion

Since ketogenic diets have been proven effective for patients with intractable epilepsy, it becomes essential for healthcare providers to help maintain ketosis to prevent relapse of seizures. Pharmacists can utilize their resources to recommend medications with low-carbohydrate preparations for patients on a ketogenic diet. The general rule is that there is a greater carbohydrate content in liquid formulations than chewable and disintegrating tablets, with the least carbohydrate content found in tablets and capsules. Once physicians have initiated patients on a ketogenic diet, initiating a protocol for pharmacy to follow carbohydrate content in medications would be an excellent step towards minimizing errors in the management of ketosis.

## Figures and Tables

**Box 1:**
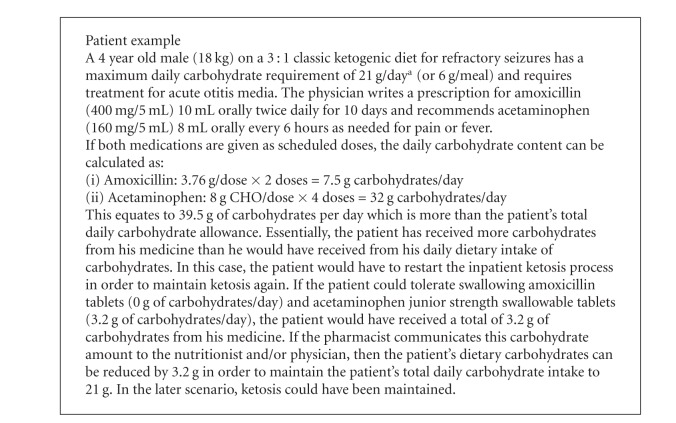
^
a^A sample calculation of the patient's daily carbohydrate requirements is provided in Table 2.

**Table 1:** Types of ketogenic diets [4].

	Macronutrient content (% total daily calories)			Comments
	Fat	Protein	Carbohydrate
				(i) 4 : 1 or 3 : 1 (fat : nonfat) ratio
Classic ketogenic diet	LCT: 85–90	6–8	2–4	(ii) Unpalatable → poor compliance
				(iii) GI effects: constipation

				(i) ~3 : 1 (fat : nonfat) ratio
MCT diet	MCT: 71	10	19	(ii) Easier to prepare
				(iii) Greater flexibility with protein and carbohydrate allowance
				(iv) GI effects: nausea, vomiting, diarrhea in ~50% patients

Modified MCT diet	LCT: 40–50	10–20	5–10	(i) Incorporates LCT and MCT
	MCT: 30			(ii) Fewer GI effects

				(i) No fasting or hospital stay
Modified Atkins diet	60–70	20–30	5	(ii) No calorie restrictions
				(iii) Less dietitian support

Low-glycemic-index treatment diet	60–70	20–30	10	(i) Only low-glycemic-index carbohydrates allowed for 10% daily carbohydrates
				(ii) Details of how diet is prescribed are not widely known [1]

LCT: long-chain triglycerides; MCT: medium-chain triglycerides; GI: gastrointestinal.

**Table 2:** Sample calculations of daily energy requirements for the 3 : 1 classic ketogenic diet for a 18 kg patient.

Daily caloric requirement	
(i) Total body weight × 68 cal/kg/day	
(ii) 18 kg × 68 cal/kg/day = 1224 cal/day	
Daily number of dietary units	
(i) For 3 : 1 (fat : protein/carbohydrate)	
(a) 3 g fat/unit × 9 cal/g fat → 27 calories	
(b) 1 g protein or CHO/unit × 4 cal/g protein or carbohydrate (CHO) = 4 calories	
(c) 27 + 4 = 31 calories/unit	
(ii) Daily caloric requirement ÷ calories/unit = dietary units/day	
(a) 1224 ÷ 31 = 39 units/day	
Daily fat content	
(i) Dietary units/day × g fat/unit = g fat/day	
(ii) 39 units/day × 3 g fat/unit = 117 g fat/day	
Daily protein and CHO content (combined)	
(i) Dietary units/day × g protein or CHO/unit = g protein or CHO/day	
(ii) 39 units/day × 1 g protein or CHO/unit = 39 g protein and CHO/day	
Daily protein content = 1 g/kg/day	
(i) 1 g/kg/day × 18 kg = 18 g/day	
Daily carbohydrate content	
(i) Combined protein and CHO content − daily protein content = daily carbohydrate content	
(ii) 39 g protein and CHO/day − 18 g protein/day = 21 g CHO/day	
Divide allotment into 3 meals	
(i) Fat: 117 ÷ 3 = 39 g/meal	
(ii) Protein: 18 ÷ 3 = 6 g/meal	
(iii) CHO: 21 ÷ 3 = 7 g/meal	

**Table 3:** Carbohydrate content in pediatric antiepileptic medications and daily total carbohydrate estimates for a five-year-old child (weighing 18 kg) on maximum monotherapy of antiepileptic drugs treatment doses (concerns for the patient on a ketogenic diet) [5]^a^. Medications with high carbohydrate content (≥2 g per dose) are highlighted in bold.

Antiepileptic medication	Dosage unit	Grams carbohydrate per dosage unit	Maximum daily dose for an 18 kg 5-year-old child	Daily carbohydrate total from maximum dosing of medication for a 18 kg 5-year-old child (grams)
Carbamazepine ** suspension (TEGretol)**	**100 mg/5 mL**	**2.65**	**35 mg/kg/day**	**16.7**
Carbamazepine chewable tablets (TEGretol)	100 mg	0.28	35 mg/kg/day	1.8
Carbamazepine tablets (TEGretol)	200 mg	0.06	35 mg/kg/day	0.2
ClonazePAM tablets (KlonoPIN)	2 mg	0.14	0.2 mg/kg/day	0.3
**Ethosuximide syrup (Zarontin)**	**250 mg/5 mL**	**3.63**	**1.5 g/day**	**21.8**
Ethosuximide capsules (Zarontin)	250 mg	0.13	1.5 g/day	0.78
Felbamate solution (Felbatol)	600 mg/5 mL	1.5	45 mg/kg/day	2
Felbamate tablets (Felbatol)	400 mg	0.13	45 mg/kg/day	0.3
Felbamate tablets (Felbatol)	600 mg	0.19	45 mg/kg/day	0.3
Gabapentin tablets (Neurontin)	100 mg	0.03	40 mg/kg/day	0.2
Gabapentin tablets (Neurontin)	300 mg	0.07	40 mg/kg/day	0.2
Gabapentin tablets (Neurontin)	400 mg	0.1	40 mg/kg/day	0.2
LamoTRIgine tablets (LaMICtal)	25 mg	0.03	10 mg/kg/day	0.2
LamoTRIgine tablets (LaMICtal)	100 mg	0.11	10 mg/kg/day	0.2
LamoTRIgine tablets (LaMICtal)	150 mg	0.16	10 mg/kg/day	0.2
LamoTRIgine tablets (LaMICtal)	200 mg	0.14	10 mg/kg/day	0.1
LamoTRIgine chewable/dispersible tablets (LaMICtal)	5 mg, 25 mg	0	10 mg/kg/day	0
Levetiracetam oral solution (Keppra)	100 mg/mL	0.3	10 mg/kg/day	0.5
Phenobarbital elixir *0.71 g ethyl alcohol/5 mL	**20 mg/5 mL**	**3.4**	**5 mg/kg/day**	**15.3**
Phenobarbital tablets	15 mg	0.06	5 mg/kg/day	0.4
Phenobarbital tablets	30 mg	0.07	5 mg/kg/day	0.2
Phenobarbital tablets	60 mg	0.1	5 mg/kg/day	0.2
Phenytoin suspension (Dilantin)	125 mg/5 mL	1.39	8 mg/kg/day	1.6
Phenytoin infatabs (Dilantin)	50 mg	0.48	8 mg/kg/day	1.4
Phenytoin kapseal (Dilantin)	30 mg	0.15	8 mg/kg/day	0.7
Phenytoin kapseal (Dilantin)	100 mg	0.11	8 mg/kg/day	0.2
Primidone oral suspension (Mysoline)	250 mg/5 mL	0	25 mg/kg/day	0
Primidone tablets (Mysoline)	50 mg	0.03	25 mg/kg/day	0.27
Primidone tablets (Mysoline)	250 mg	0.03	25 mg/kg/day	0.1
Sodium divalproex sprinkle capsules (Depakote)	125 mg	0.05	60 mg/kg/day	0.4
Sodium divalproex tablets (Depakote)	125 mg	0.03	60 mg/kg/day	0.3
Sodium divalproex tablets (Depakote)	250 mg	0.05	60 mg/kg/day	0.2
Sodium divalproex tablets (Depakote)	500 mg	0.1	60 mg/kg/day	0.2
Topiramate tablets (Topamax)	25 mg	0.04	9 mg/kg/day	0.3
Topiramate tablets (Topamax)	100 mg	0.17	9 mg/kg/day	0.3
Topiramate tablets (Topamax)	200 mg	0.09	9 mg/kg/day	0.1
**Valproic acid syrup (Depakene)**	**250 mg/5 mL**	**4.5**	**60 mg/kg/day**	**19.4**
Valproic acid capsules (Depakene)	250 mg	0	60 mg/kg/day	0

^a^For a five-year-old child weighing 18 kg, the maximum recommended carbohydrate amount is 21 g per day for the 3 : 1 classic ketogenic diet.

**Table 4:** Carbohydrate content in pediatric analgesics/antipyretics and daily total carbohydrate estimates for a five-year-old child (weighing 18 kg) on maximum treatment doses (concerns for the patient on a ketogenic diet) [5]^a^. Medications with high carbohydrate content (≥2 g per dose) are highlighted in bold.

Description (brand name)	Dosage unit	Grams carbohydrate per dosage unit	Maximum daily dose for an 18 kg 5-year-old child	Daily carbohydrate total from maximum dosing of medication for a 18 kg 5-year-old child (grams)
Acetaminophen extended release caplets (Tylenol)	650 mg	<0.03	15 mg/kg/dose for 5 doses/day	<1
Acetaminophen extra strength caplets (Tylenol)	500 mg	<0.05	15 mg/kg/dose for 5 doses/day	<1
Acetaminophen extra strength gel caps (Tylenol)	500 mg	<0.05	15 mg/kg/dose for 5 doses/day	<1
Acetaminophen infant drops (grape and cherry) (Tylenol)	0.8 mL	<0.71	15 mg/kg/dose for 5 doses/day	n/a for this patient
**Acetaminophen liquid suspension (cherry) (Tylenol)**	**160 mg/5 mL**	**<5**	15 mg/kg/dose for 5 doses/day	**42.2**
Acetaminophen regular strength caplets (Tylenol)	325 mg	<0.04	15 mg/kg/dose for 5 doses/day	<1
Acetaminophen elixir (Tylenol)	160 mg/5 mL	<1.6	15 mg/kg/dose for 5 doses/day	13.5
**Acetaminophen extra strength liquid (Tylenol)**	**1000 mg/30 mL**	**<5.7**	15 mg/kg/dose for 5 doses/day	**7.7**
Acetaminophen junior strength swallowable caplets (Tylenol)	160 mg	<0.4	15 mg/kg/dose for 5 doses/day	<1
**Acetaminophen grape flavored suspension (Tylenol)**	**160 mg/5 mL**	**<4.8**	15 mg/kg/dose for 5 doses/day	**40.5**
**Acetaminophen elixir with codeine (Tylenol with Codeine)** *0.35 g ethyl alcohol/5 mL	**120 mg/5 mL**	**3**	15 mg/kg/dose of APAP^b^ for 5 doses/day	**33.8**
Acetaminophen with codeine tablets (Tylenol with Codeine)	All strengths	0.05	15 mg/kg/dose of APAP for 5 doses/day	<1
Acetaminophen chewable tablets (Tylenol)	80 mg	0.25	15 mg/kg/dose for 5 doses/day	4
Ibuprofen tablets (Advil)	200 mg	0.23	40 mg/kg/day	0.7
Ibuprofen drops (Motrin)	40 mg/mL	<0.41	40 mg/kg/day	7.4
Ibuprofen suspension (Motrin)	100 mg/5 mL	<0.63	40 mg/kg/day	4.5
Ibuprofen chewable tablets (Motrin)	50 mg	<0.28	40 mg/kg/day	3.9
Ibuprofen chewable tablets (Motrin)	100 mg	<0.54	40 mg/kg/day	3.8

^a^For a five-year-old child weighing 18 kg, the maximum recommended carbohydrate amount is 21 g per day for the 3 : 1 classic ketogenic diet.

^b^APAP: N-acetyl-para-aminophenol, or better known as acetaminophen.

**Table 5:** Carbohydrate content in pediatric antibiotic medications and daily total carbohydrate estimates for a five-year-old child (weighing 18 kg) on maximum recommended treatment doses (concerns for the patient on a ketogenic diet) [5]^a^. Medications with high carbohydrate content (≥2 g per dose) are highlighted in bold.

Antibiotics	Dosage unit	Grams carbohydrate per dosage unit	Maximum daily dose for an 18 kg 5-year-old child	Daily carbohydrate total from maximum dosing of medication for a 18 kg 5-year-old child (grams)
Amoxicillin pediatric drops (Amoxil)	50 mg/mL	1.6	100 mg/kg/day	n/a for this patient
Amoxicillin oral suspension (Amoxil)	125 mg/5 mL	1.7	100 mg/kg/day	24.5
Amoxicillin oral suspension (Amoxil)	250 mg/5 mL	1.85	100 mg/kg/day	13.3
Amoxicillin oral suspension (Amoxil)	400 mg/5 mL	1.88	100 mg/kg/day	8.46
Amoxicillin chewable tablets (Amoxil)	125 mg	0.05	100 mg/kg/day	0.7
Amoxicillin chewable tablets (Amoxil)	250 mg	0.34	100 mg/kg/day	2.4
Amoxicillin capsules (Amoxil)	250 mg	0	100 mg/kg/day	0
Amoxicillin capsules (Amoxil)	500 mg	0	100 mg/kg/day	0
**Amoxicillin oral suspension (Trimox)**	**125 mg/5 mL**	**3.3**	100 mg/kg/day	**47.5**
**Amoxicillin oral suspension (Trimox)**	**250 mg/5 mL**	**3.3**	100 mg/kg/day	**23.8**
Amoxicillin capsules (Trimox)	250 mg	0	100 mg/kg/day	0
Amoxicillin capsules (Trimox)	500 mg	0	100 mg/kg/day	0
Amoxicillin/clavulanate potassium oral suspension (Augmentin)	125 mg/5 mL	0.52	40 mg/kg/day amoxicillin component	3
Amoxicillin/clavulanate potassium oral suspension (Augmentin)	200 mg/5 mL	0.06	45 mg/kg/day amoxicillin component	0.2
Amoxicillin/clavulanate potassium oral suspension (Augmentin)	250 mg/5 mL	0.6	45 mg/kg/day amoxicillin component	1.9
Amoxicillin/clavulanate potassium oral suspension (Augmentin)	400 mg/5 mL	0.06	100 mg/kg/day amoxicillin component	0.3
Amoxicillin/clavulanate potassium chewable tablets (Augmentin)	125 mg	0.08	45 mg/kg/day amoxicillin component	0.5
Amoxicillin/clavulanate potassium chewable tablets (Augmentin)	250 mg	0.34	45 mg/kg/day amoxicillin component	1
Amoxicillin/clavulanate potassium chewable tablets (Augmentin)	400 mg	0.36	100 mg/kg/day amoxicillin component	1.6
Amoxicillin/clavulanate potassium tablets (Augmentin)	250 mg	0.02	45 mg/kg/day amoxicillin component	<1
Amoxicillin/clavulanate potassium tablets (Augmentin)	500 mg	0.02	45 mg/kg/day amoxicillin component	<1
Amoxicillin/clavulanate potassium tablets (Augmentin)	875 mg	0.03	100 mg/kg/day amoxicillin component	<1
**Ampicillin oral suspension (Omnipen)**	**125 mg/5 mL**	**4**	**100 mg/kg/day**	**57.6**
**Ampicillin oral suspension (Omnipen)**	**250 mg/5 mL**	**4**	**100 mg/kg/day**	**28.8**
**Azithromycin oral suspension (Zithromax)**	**100 mg/5 mL**	**3.86**	**10 mg/kg/day**	**6.9**
Azithromycin tablets (Zithromax)	250 mg	0.06	10 mg/kg/day	<1
**Cefaclor oral suspension (Ceclor)**	**125 mg/5 mL**	**2.95**	**40 mg/kg/day**	**17**
**Cefaclor oral suspension (Ceclor)**	**187 mg/5 mL**	**2.83**	**40 mg/kg/day**	**10.9**
**Cefaclor oral suspension (Ceclor)**	**250 mg/5 mL**	**2.83**	**40 mg/kg/day**	**8.2**
**Cefaclor oral suspension (Ceclor)**	**375 mg/5 mL**	**2.6**	**40 mg/kg/day**	**5**
Cefaclor pulvules (Ceclor)	250 mg	0.04	40 mg/kg/day	<1
Cefaclor pulvules (Ceclor)	500 mg	0.07	40 mg/kg/day	<1
**Cefadroxil oral suspension (Duricef)**	**250 mg/5 mL**	**3**	**30 mg/kg/day**	**6.5**
**Cefadroxil oral suspension (Duricef)**	**125 mg/5 mL**	**3.1**	**30 mg/kg/day**	**13.4**
Cefadroxil capsules (Duricef)	500 mg	0.13	30 mg/kg/day	0.13
Cefadroxil film-coated tablets (Duricef)	1 g	0.13	30 mg/kg/day	<1
**Cefixime oral suspension (Suprax)**	**100 mg/5 mL**	**2.7**	**8 mg/kg/day**	**3.9**
Cefixime tablets (Suprax)	200 mg	0.06	8 mg/kg/day	n/a
Cefixime tablets (Suprax)	400 mg	0.12	8 mg/kg/day	n/a
**Cefpodoxime proxetil oral suspension (Vantin)**	**50 mg/5 mL**	**3**	**10 mg/kg/day**	**10.8**
**Cefpodoxime proxetil oral suspension (Vantin)**	**100 mg/5 mL**	**3.05**	**10 mg/kg/day**	**5.5**
Cefpodoxime proxetil tablets (Vantin)	100 mg	0.04	10 mg/kg/day	<1
Cefpodoxime proxetil tablets (Vantin)	200 mg	0.08	10 mg/kg/day	0.08
**Cefprozil oral suspension (Cefzil)**	**125 mg/5 mL**	**2**	**20 mg/kg/day**	**5.8**
Cefprozil oral suspension (Cefzil)	250 mg/5 mL	1.9	20 mg/kg/day	2.7
Cefprozil tablets (Cefzil)	250 mg	0.02	20 mg/kg/day	<0.1
Cefprozil tablets (Cefzil)	500 mg	0.03	20 mg/kg/day	<0.1
**Cefuroxime axetil suspension (Ceftin)**	**125 mg/5 mL**	**3.23**	**30 mg/kg/day**	**14**
Cefuroxime axetil tablets (Ceftin)	125 mg	0	30 mg/kg/day	0
Cefuroxime axetil tablets (Ceftin)	250 mg	0	30 mg/kg/day	0
Cefuroxime axetil tablets (Ceftin)	500 mg	0	30 mg/kg/day	0
**Cephalexin oral suspension (Keflex)**	**125 mg/5 mL**	**3.13**	**100 mg/kg/day**	**45.1**
**Cephalexin oral suspension (Keflex)**	**250 mg/5 mL**	**3.03**	**100 mg/kg/day**	**21.8**
Cephalexin pulvules (Keflex)	250 mg	0.13	100 mg/kg/day	0.9
Cephalexin pulvules (Keflex)	500 mg	0.13	100 mg/kg/day	0.4
Ciprofloxacin tablets (Cipro)	250 mg	0.04	30 mg/kg/day	0.1
Ciprofloxacin tablets (Cipro)	500 mg	0.07	30 mg/kg/day	0.07
Ciprofloxacin tablets (Cipro)	750 mg	0.11	30 mg/kg/day	0.11
Ciprofloxacin oral suspension (Cipro)	250 mg/5 mL	1.4	30 mg/kg/day	3
Ciprofloxacin oral suspension (Cipro)	500 mg/5 mL	1.3	40 mg/kg/day	1.4
**Clarithromycin suspension (Biaxin)**	**125 mg/5 mL**	**3**	**15 mg/kg/day**	**6.5**
**Clarithromycin suspension (Biaxin)**	**250 mg/5 mL**	**2.3**	**15 mg/kg/day**	**2.5**
Clarithromycin tablets (Biaxin)	250 mg	0.07	15 mg/kg/day	0.07
Clarithromycin tablets (Biaxin)	500 mg	0	15 mg/kg/day	0
Erythromycin base tablets (Ery-Tab)	333 mg	0	50 mg/kg/day	0
Erythromycin base tablets (Ery-Tab)	500 mg	0	50 mg/kg/day	0
Erythromycin estolate oral suspension (Ilosone)	125 mg/5 mL	1.85	50 mg/kg/day	13.3
Erythromycin estolate oral suspension (Ilosone)	250 mg/5 mL	1.8	50 mg/kg/day	6.5
Erythromycin estolate pulvules (Ilosone)	250 mg	0	50 mg/kg/day	0
Erythromycin estolate tablets (Ilosone)	500 mg	0.11	50 mg/kg/day	0.2
Erythromycin ethylsuccinate drops (EryPed)	10 mg/2.5 mL	1.5	50 mg/kg/day	n/a
Erythromycin ethylsuccinate chewable tablets (EryPed)	200 mg	1.44	50 mg/kg/day	6.5
**Erythromycin ethylsuccinate suspension (E.E.S.)**	**200 mg/5 mL**	**3.5**	**50 mg/kg/day**	**15.8**
**Erythromycin ethylsuccinate suspension (E.E.S.)**	**400 mg/5 mL**	**3.5**	**50 mg/kg/day**	**7.9**
Erythromycin ethylsuccinate granules (E.E.S.)	200 mg/5 mL	1.5	50 mg/kg/day	6.8
Erythromycin ethylsuccinate filmtabs (E.E.S.)	400 mg	0.2	50 mg/kg/day	0.4
Erythromycin ethyl + sulfisoxazole acetyl suspension (Pediazole)	200 mg/5 mL	1.9	50 mg/kg/day	8.6
Erythromycin ethyl + sulfisoxazole acetyl suspension (Pediazole)	600 mg/5 mL	1.9	50 mg/kg/day	2.9
Nitrofurantoin oral suspension (Furadantin)	25 mg/5 mL	0.7	7 mg/kg/day	3.5
**Penicillin V potassium oral suspension**	**125 mg/5 mL**	**2.53**	**50 mg/kg/day**	**18.2**
**Penicillin V potassium oral suspension**	**250 mg/5 mL**	**3.28**	**50 mg/kg/day**	**11.8**
Penicillin V potassium tablets	250 mg	0.09	50 mg/kg/day	0.3
Penicillin V potassium tablets	500 mg	0	50 mg/kg/day	0
**Trimethoprim (TMP) and sulfamethoxazole (SMX) suspension (Septra)**	**40 mg** **TMP/200 mg** **SMX/5 mL**	**2.35**	**20 mg/kg/day** **TMP component**	**21.2**
**Trimethoprim (TMP) and sulfamethoxazole (SMX) grape suspension (Septra)**	**40 mg** **TMP/200 mg** **SMX/5 mL**	**2.35**	**20 mg/kg/day** **TMP component**	**21.2**
Trimethoprim (TMP) and sulfamethoxazole (SMX) tablets (Septra)	80 mg TMP/400 mg SMX/5 mL	0	20 mg/kg/day TMP component	0
Trimethoprim (TMP) and sulfamethoxazole (SMX) double strength tablets (Septra)	160 mg TMP/800 mg SMX/5 mL	0	20 mg/kg/day TMP component	0

^a^For a five-year-old child weighing 18 kg, the maximum recommended carbohydrate amount is 21 g per day for the 3 : 1 classic ketogenic diet.
